# Cultural influences on road safety in Vanuatu: masculinity norms and their impact on dangerous driving behaviors

**DOI:** 10.3389/fpubh.2025.1715906

**Published:** 2025-11-14

**Authors:** Saen Fanai, Eunice Okyere, Kissinger Marfoh

**Affiliations:** 1 College of Medicine, Nursing and Health Science, Fiji National University, Suva, Fiji; 2 School of Public Health, College of Medicine, Nursing and Health Science, Fiji National University, Suva, Fiji

**Keywords:** masculinity norms, dangerous driving behaviors, road safety, behavior change interventions, Vanuatu

## Abstract

Road traffic injuries are a growing public health concern in Vanuatu, mirroring global trends where dangerous driving behaviors such as speeding, distracted driving, and drunk driving contribute significantly to accidents and injuries. Driving in Vanuatu is culturally perceived as a male-dominated activity. This study examines how cultural factors, including norms of masculinity, influence driving practices and their impact on road safety. Understanding these influences is crucial for designing effective behavior change interventions aimed at reducing unsafe driving behaviors. The study employed qualitative methods, including in-depth interviews with drivers (*n* = 9), community leaders (*n* = 10), and focus group discussions with law enforcement officers (*n* = 10). The data collection methods employed allowed the researchers to gather detailed, context-specific insights on the main themes observed. Study participants were recruited through purposive sampling. Data were analyzed through thematic analysis, uncovering culturally rooted patterns that influence road use. Three key themes emerged: First, masculinity norms contribute to dangerous driving behaviors in Vanuatu, with men frequently speeding, drink-driving, and engaging in other risky driving actions to prove bravery. Society expects men to operate commercial and public transportation, while women are viewed as less capable. Second, community perceptions of road traffic injuries blame fate, divine will, inexperienced drivers, poor road conditions, and weak enforcement. Third, cultural norms as barriers to safe driving highlight how traditional beliefs and community acceptance of risky behaviors, such as drink-driving during celebrations, normalize unsafe practices and hinder road safety efforts. The driving culture of Vanuatu significantly mirrors traditional masculinity standards, which contribute to hazardous driving practices. Effective behavior changes interventions depend on culturally adapted, community-led programs, including peer education targeting young men and initiatives that promote women’s participation in public transport roles. To achieve sustainable road safety improvements, interventions must be co-designed with communities, challenge harmful gender expectations, and be supported by consistent enforcement strategies.

## 1 Introduction

Road traffic injury (RTI) is responsible for the deaths of approximately 3,700 individuals globally each day and around 1.35 million each year, positioning them among the top causes of death for those aged 5 to 29 (1, 2). Individuals aged 15 to 44, both youth and adults, who frequently support their families, constitute over half of those injured or killed in road traffic accidents (3). Over 50% of road traffic fatalities are among vulnerable road users such as pedestrians, cyclists, and motorcyclists (1). The factors contributing to road traffic injuries (RTIs) differ across different parts of the globe. In low- and middle-income countries (LMIC), such as those in the Pacific region, issues like rapid urban growth coupled with poorly designed and unsafe road systems, unsuitable or unsafe vehicles, inadequate enforcement of traffic regulations, reckless driving, and the lack of timely and effective pre-hospital care for RTI victims are among the numerous risk factors for RTIs (4, 5). The findings of a handful of studies carried out in the Pacific region on RTIs reveal that the majority of these incidents stem from vehicle collisions linked to human error, with key risk factors including speeding, driving while intoxicated by alcohol and other mind-altering substances, reckless driving, and driving when fatigued, and the negligence of using motorcycle helmets and seatbelts (3, 6–8). This highlights a responsibility failure on the part of drivers or those operating the vehicles. While recent studies have explored gender and driving behavior globally (9, 10), few have examined how masculinity and cultural expectations shape risky driving within Pacific Island contexts.

Most response initiatives aimed at tackling road traffic injuries tend to focus on law enforcement, infrastructure improvements, and pre-hospital care. These approaches are less effective in influencing driver behavior and are costly and challenging to implement in our regions (3). To combat road traffic injuries in the Pacific, particularly in Vanuatu, any preventive strategies should prioritize behavioral change interventions for drivers, passengers, and pedestrians (11). Various models and strategies for behavioral change interventions can address a range of behavior-related concerns (12). Findings from extensive randomized studies on behavior change interventions suggest that implementing the right models and strategies can significantly influence behavior change in specific populations, leading to a notable decrease in RTIs. Additionally, these studies have shown that certain information, education, and communication approaches are cost-effective (13).

Driver behavior is a central determinant of crash risk. Efforts to reduce road traffic injuries increasingly recognize that interventions must be grounded in the social and cultural realities of the communities they serve (6). Very few studies in the Pacific region have tried to explore the cultural factors behind speeding, impaired driving, distracted driving, and driver fatigue, all of which contribute to road traffic injuries (11). Developing effective road safety strategies now requires creating interventions that consider the social and cultural contexts of the communities involved (3, 6). Evidence-based behavior change interventions that overlook local cultural norms are unlikely to gain community support (14, 15). The success of strategies like helmet promotion, speed-limit enforcement, and drunk-driving deterrence relies on their ability to resonate with local beliefs, values, and social norms. Interventions that lack knowledge of driving culture, risk-taking behaviors, and gender roles may appear forced to local communities, making it difficult to change deeply ingrained driving habits (1).

The cultural context of countries in the Pacific requires special consideration when developing BCIs, due to individual countries’ communal decision-making processes and traditional leadership structures (13). The cultural values of masculinity, social standing, and group identity influence driving behavior and how communities respond to road safety messages (13, 16). Research indicates that gender-based social expectations act as risk factors that raise the likelihood of road traffic injuries worldwide (6). Multiple studies across Europe, Asia, and the Americas show that men are more likely to engage in dangerous driving behaviors than women, and they constitute the majority of fatal road accidents (1, 6).

The unique social environment of Vanuatu warrants a more detailed examination of how its cultural elements influence driving habits and road safety outcomes, particularly in light of the interaction between traditional masculine norms, community decisions, and inadequate law enforcement systems. Developing effective community-based road safety initiatives requires understanding cultural factors that contribute to risky behaviors, as these programs must challenge harmful gender stereotypes.

Understanding these cultural dynamics clarifies why dangerous driving persists and highlights strategies, such as engaging community leaders and challenging gender stereotypes, that can underpin effective, long-term road safety programs.

### 1.1 Aim

This research examined how cultural elements, especially masculinity norms, influence dangerous driving behavior in Vanuatu. The study sought to:

- Explore community and driver perceptions of gender roles and their impact on driving habits.
- Explore how traditional views of masculinity influence men’s risky driving behaviors.
- Offers valuable local cultural insights to inform effective road safety policies and programs.

## 2 Methodology

### 2.1 Study design

This qualitative study employed in-depth interviews (IDIs) and focus group discussions (FGDs). IDIs were used for vehicle drivers and Community leaders (CLs), and FGDs were used for law enforcement officers (LEOs). This qualitative study design enabled the researcher to gain deep insights into the cultural acceptance, feasibility, and practical application of behavior change interventions, as well as the potential challenges and enablers for their implementation. The use of this qualitative research approach allowed the researcher to understand people’s ideas in their natural settings, aiming to make sense of the phenomena through the meanings people attribute to them (17–19).

Through FGD, information was provided more naturally and quickly than if people were interviewed separately (20). The FGD for LEOs provided a broader range of information and allowed for the opportunity to seek clarification on topics that required further understanding (21). To ensure shared representation and equal participation, the focus group discussion was conducted with the LEO group, which shared similar traits. This allowed them to feel more comfortable expressing themselves freely (20, 21).

On the other hand, IDI utilized unstructured, direct, and personal interview questions directed at vehicle drivers and community leaders. In this process, the researcher began with a broad question, inviting the respondents to openly express their opinions on the phenomenon (22). This yielded very rich information and provided the chance to ask follow-up questions, probe for additional details, justify earlier responses, and establish links between various topics. It also created a comfortable atmosphere where participants felt more comfortable engaging in conversation (21, 22).

### 2.2 Study population and sample

The study population comprised community leaders (CLs), including chiefs, youth leaders, and church leaders; LEOs, such as police officers, municipal wardens, and public land transport personnel; and drivers who were private, public, and commercial vehicle drivers (PPCVDs) who met the inclusion and exclusion criteria listed in Table 1. Research indicates that involving community leaders in programs designed to change high-risk individuals’ lifestyle behaviors is achievable and positively received, based on community-level perspectives (23). LEO’s perspectives on preventing road traffic injuries have effectively tackled unsafe driving behavior (24, 25). Furthermore, studies indicate that PPCVDs frequently witness and respond to RTI incidents, yet their perspectives on preventing such incidents are often overlooked despite their importance (11, 26). These are important stakeholders and informants on RTI in Vanuatu. All study participants were recruited from Port Vila, the capital city of Vanuatu.

**Table 1:** Inclusion and exclusion criteria.

Target group	Inclusion criteria	Exclusion criteria
Law Enforcement Officers (LEOs), including Municipal wardens and the Public Land Transport Authority personnel	More than 6 months of working experience Male and Female Aged 18 and over Working in Port Vila Self-identified as a Vanuatu citizen	Medically unfit to participate Unwilling to participate in the study
Public, Private, and Commercial Vehicle drivers (PPCVDs)	Bus and taxi drivers Private Vehicle drivers Aged between 18 and over Male and Female More than 6 months in the land transport industry Working in Port Vila	Medically unfit to participate Unwilling to participate in the study
Community Leaders (CLs) who are: Chiefs Youth leaders Church leaders	Male and female Current youth leader Current church leader or leader of a faith-based organization Currently a chief, or a community leader Resident of Port Vila Must be 25 years or older Was in the current leadership role for over a year Port Vila resident for over 6 months	Medically unfit to participate Unwilling to participate

### 2.3 Study setting

The study occurred in Port Vila, the capital and largest city of Vanuatu. Located on the island of Efate, Port Vila has an estimated population of 60,076 (27). Most residents of Port Vila are Melanesian, though there are also small communities of Polynesians, Asians, Australians, and Europeans, with the French and British being the most prominent (28). Currently, there are 3,529 registered vehicles in Vila; however, this number may not be entirely accurate, as the database has not been updated, and privately owned vehicles may be operating illegally. Furthermore, there are 491 commercial and public transport vehicles, including minibusses, taxis, and pick-up trucks (29).

PPCVDs were accessed at the leading taxi and transport base, supermarkets, homes, and public parking areas. CLs, comprising youth leaders, chiefs, and church leaders, were contacted at their respective administrative locations, including the youth centers, the chief’s nakamal, and the church centers. The LEOs were engaged at their respective police posts, including the main police headquarters.

### 2.4 Sampling and sample size

Purposive sampling was employed for this study to recruit 10 CLs and 9 PPCVDs for the IDIs, and 10 LEOs for FGD. This method is cost-effective and time-efficient, making it suitable for this study component due to the limited number of primary data sources contributing to this part of the research (30). Furthermore, this approach is practical for exploring the general perception of people’s behaviors and situations from an intuitive perspective (30, 31).

Between October and November 2024, 10 CLs and 9 PPCVDs participants were recruited for the IDIs. Two FGDs were conducted among LEOS, including police, municipal wardens, and public land transport authority personnel. The participant numbers for the IDIs and FGDs were determined based on data saturation (30).

### 2.5 Data collection technique and instruments

The study employed semi-structured interviews, where the interviewer referred to guiding questions but had the flexibility to adapt and add questions based on the participants’ responses and context, allowing for more intuitive and natural conversations (30). The interviewer audio-recorded the discussion with the participants’ consent, which was later transcribed. Qualitative research requires recording interviews, as it helps researchers preserve participants’ exact statements, thus maintaining the authenticity and integrity of the data (32). By using recordings, researchers can focus entirely on the interview conversation without the distraction of note-taking (30). The reliability of recordings as a source for transcription and their ability to support detailed data analysis contribute to more credible findings, which enhance trustworthiness (32). The FGDs involved discussions with LEOs to explore their experiences, perceptions, knowledge, and the reasons behind their thoughts on how people behave in specific ways. The two FGDs involved 10 LEOs, with 4 and 6 LEOs in each FGD, and were facilitated by the researcher using the established topic guide. The interview guide compiled the questions that the interviewer planned to cover during the interview (30). The discussions were audio-recorded with the participant’s permission and were later transcribed. The FGDs enabled the rapid collection of information from a diverse group of LEOs, who represented the views of community LEOs, supplementing the IDIs (20).

### 2.6 Study procedure

Ethical approval was obtained from FNU’s Human Health Research & Ethics Committee (FNU HHREC) and the Vanuatu Ministry of Health Research and Ethics Committee. Formal communication was established with relevant department heads to inform and identify their officers for participation in recruiting LEOs. The researcher independently recruited bus and taxi drivers at public transportation centers and sub-centers.

Participants were required to agree to participate before the interviews and discussions took place. They received information sheets translated into Bislama and were then oriented on the study’s purpose. Written consent was mandatory for this study; therefore, participants who agreed were asked to complete and sign a consent form, translated into Bislama. The confidentiality of our participants was assured, enabling them to provide information without bias.

In-depth interviews (IDIs) for PPCVDs and CLs were conducted at public transport centers and sub-centers, as well as at churches, youth centers, and Nakamals, according to the convenience of the participants. The leading researcher ensured that all in-depth face-to-face interviews took place with other potential study participants or colleagues who might influence responses (20). Similarly, FGDs were organized to ensure that all participants agreed on a place and time for the discussion. Ideally, all FGDs were conducted at their respective workplaces. Participants were contacted individually to suggest and confirm the most suitable time for the FGD.

The IDI questions began with demographic information and then proceeded to the semi-structured questions. For FGDs, a guide was used to facilitate the discussions. Additionally, a questionnaire for demographic information, translated into Bislama, was distributed to participants, and written responses were collected at the end of the FGD. Throughout the interviews and discussions, probing techniques were employed, based on each participant’s reflections and guided by predetermined questions. After each interview or discussion, preliminary data analysis was conducted concurrently to identify ideas for the following interview or discussion (30). All interviews and discussions were held in Bislama, the common language of Vanuatu.

Throughout the study, the researcher conducted a maximum of four IDIs per day to prevent exhaustion, which could negatively impact the quality of the questions asked and the richness of the data (30, 33). Each interview lasted 20 to 25 min, while the FGDs took approximately 40 to 50 min. All interviews and FGDs were recorded with a voice recorder. Verbal consent and permission to audio-record the discussions were obtained from participants (32). Data collection through both in-depth interviews and FGDs continued until saturation of each concept was achieved, at which point further data collection no longer introduced new information, and no new codes or themes emerged after three consecutive interviews. Peer debriefing between the lead researcher and two independent reviewers was used to verify coding consistency and theme validity (30, 32).

### 2.7 Study rigor

This study employed purposive sampling to select participants with significant experience in RTI issues, ensuring credibility (34). Triangulation was achieved by utilizing data from various sources, including in-depth interviews and focus groups, to capture the participants’ perspectives (32, 34). To ensure transferability, participants were selected to represent diverse social backgrounds, including those from various genders and transportation stakeholders from the broader population (34). Dependability was ensured by maintaining a clear audit trail and thoroughly documenting the research process, which included data collection, recording interviews, coding, and analysis. Reflexivity was also incorporated by acknowledging potential biases and making them explicit in the data analysis and interpretation (34). Finally, two independent researchers conducted a peer debriefing to enhance confirmability, reviewing and questioning the findings (32). During the study, the researcher maintained a reflective log to record thoughts and conclusions, ensuring these were grounded in the participants’ narratives rather than the researcher’s assumptions (32, 34).

### 2.8 Data management and analysis

The qualitative data were analyzed using thematic analysis, using Braun and Clarke’s established framework for identifying, analyzing, and reporting patterns in qualitative research (32). This approach provided a detailed understanding of participants’ experiences with risky driving and their cultural perspectives on road safety. Data analysis was conducted in several phases to ensure clarity, consistency, and methodological rigor.

- Phase 1: Data preparation and familiarization

All IDIs and FGDs were audio recorded, then transcribed verbatim and checked for accuracy. Researchers carefully read the transcripts several times to become immersed, noting initial impressions and recurring ideas, which helped deepen their understanding of the content (30, 32).

- Phase 2: Initial coding

Using an inductive approach, meaningful text segments were coded line by line. Manual coding with Microsoft Excel was used to create a systematic framework for managing the dataset. These codes captured both explicit and implicit meanings in the text, supporting the research goal (35). Initial codes were generated manually using Microsoft Excel; they were later reviewed and validated collaboratively by two researchers through peer debriefing to ensure intercoder consistency. No dedicated software (e.g., NVivo) was used.

- Phase 3: Searching for themes

Codes were analyzed to identify recurring patterns that emerged as potential themes and sub-themes covering the entire dataset. The approach allowed for understanding how cultural elements and contextual factors, such as community beliefs about road accidents and traditional masculine standards, influenced risky driving behaviors (32).

- Phase 4: Reviewing and refining themes

The coded extracts and full dataset were evaluated to ensure each theme demonstrated both coherence and distinctiveness from others. Revisions involved merging similar themes and clarifying their boundaries to reflect participant stories accurately (32).

- Phase 5: Defining, naming, and interpreting themes

Themes were clearly identified and linked to research questions and relevant literature to understand their broader implications. Representative quotations were chosen to show participant authenticity while illustrating each theme with direct quotes (32, 36).

Triangulation was achieved by comparing results from IDIs and FGDs through their audit trail. The study achieved transferability by selecting participants from diverse backgrounds and maintained dependability and confirmability through the reflective journaling process (30, 37).

### 2.9 Ethical considerations

This study received approval from the FNU Human Health Research Ethics Committee (FNU HHREC) and the Vanuatu Ministry of Health Research and Ethics Committee. Participation in this study was entirely voluntary, and all responses were anonymised to protect the confidentiality and privacy of participants. Participants were informed that their involvement was voluntary and that the information collected during the interviews and discussions would remain confidential (30).

The researcher translated the information and consent forms into Bislama. Before obtaining written consent from respondents to be interviewed and recorded, the principal researcher provided information explaining the study’s purpose orally and in writing. Participants’ views and responses were documented for data processing, with their names kept anonymous (38).

Electronic copies of the data are stored on password-protected computers and external drives, while hard copies are secured in locked filing cabinets at the Ministry of Health, Vanuatu. The dataset will be retained for 3 years before it can be destroyed (31).

## 3 Results

### 3.1 Demographic characteristics

The demographic characteristics of the participants (*N* = 29) in this study are summarized in Table 2. LEOs and Community Leaders accounted for the majority of participants, with 10 individuals in each category. Among the LEOs, the majority (*N* = 5) were police officers, while among the community leaders, the majority were chiefs (*N* = 4) and youth leaders (*N* = 4). Nine participants were vehicle drivers, with three each being public transport drivers, commercial vehicle drivers, and private vehicle drivers. Most participants were aged 41 to 50 years (*N* = 16), and 86% (*N* = 25) were male. The majority of participants (*N* = 16) had completed secondary-level education. Most participants (*N* = 9) have been driving for between 1 and 10 years, and for 11 to 20 years, respectively.

**Table 2:** Demographic characteristics of participants (*N* = 29).

Participant information	Frequency	Percentage (%)
Participant categories		
Law enforcement officers		(34%)
Police	5	17
Land Transport Authority	2	7
Municipal warden	3	10
Vehicle drivers		(30%)
Public Transport Drivers	3	10
Commercial Vehicle Drivers	3	10
Private Vehicle Drivers	3	10
Community leaders		(35%)
Chiefs	4	14
Youth Leaders	4	14
Church Leaders	2	7
Age		
21–30 years old	4	14
31–40 years old	5	17
41–50 years old	16	55
51–60 years old	4	14
Gender		
Male	25	86
Female	4	14
Education level		
No education	4	14
Primary	7	24
Secondary	16	55
Tertiary	2	7
Number of years driving		
0–10 Years	9	31
11–20 Years	9	31
21 + Years	5	17
Not driving	6	21

The predominance of male participants (86%) reflects the male-dominated nature of driving in Vanuatu, which contextualizes the theme of masculinity in subsequent analyses.

### 3.2 Themes and codes

Analysis of the IDIs and FGD data uncovered three interconnected themes that reflect the cultural aspects of dangerous driving in Vanuatu: Masculinity Norms, Community Perceptions of RTIs, and Cultural Norms as Barriers to Safe Driving (Table 3).

**Table 3:** Themes, sub-themes, and codes.

Themes	Sub themes	Codes
Masculinity Norms	Driving as a male role	Driving seen as men’s job.
	Risk-taking as proof of strength	Speeding to impress friends.
		Aggressive driving shows courage
	Perception of women as unsafe drivers and safe drivers	Women considered weak or unfit to drive
		Women as safer drivers.
Community Perceptions of RTIs	Fatalistic beliefs	RTIs seen as God’s plan.
		Victims punished for unknown reasons.
	RTI attributed to youth and weak law enforcement	Young drivers blamed for accidents.
		Lack of road signs and poor enforcement.
	Rejection of Western safety models	Western road safety activities not practical
Cultural Norms as Barriers to Safe Driving	Acceptance of drinking and driving	Drinking and driving during celebrations accepted.
		Men use reckless driving to prove masculinity.
	Community tolerance for risky driving	Community fails to report unsafe drivers
	Limited collective responsibility	Cultural methods needed for road safety education.
		Dangerous driving seen as normal.

These themes show how gender norms, shared beliefs about fate and divine will, and traditional community values influence both individual driving habits and public responses to road safety concerns. Many participants explained that driving is perceived as a male-dominated activity because men often exhibit risky driving behaviors to display strength and bravery. At the same time, many community members regarded RTIs as inevitable events, which lessened the perceived need for preventive measures. These insights reveal how deeply embedded cultural beliefs not only normalize risky driving but also pose significant challenges to behavior change initiatives aimed at improving road safety.

#### 3.2.1 Theme 1: masculinity norms

According to the participants, many people perceive driving in Vanuatu as a male job. Men drive aggressively to show competitiveness, strength, and courage. They speed to impress friends.

“*Women rarely drive commercial and public transport vehicles because driving jobs are perceived as male-oriented. It seems humorous to see a woman driving a taxi, a minibus, or a commercial vehicle.”* (YL /29 years old)

The participants believe that if people change their perspectives on women’s roles in society, issues like unsafe driving could be minimized. They think that roads would be safer if more women were to operate commercial and public transport vehicles.

*“People need to shift their mentality about driving being a man’s job. If women drive, the roads would be safer”.* (MW 46 years old)

Vanuatu is a highly male-dominated society. Men demonstrate their worthiness in different ways. According to the participants, driving aggressively such as not slowing down on curved roads or before turning is one way to show courage and strength. Drivers who speed do so to impress their friends.

*“Vanuatu is a highly male-dominated society where traditional views on masculinity often see risky driving behaviors, like drunk driving and speeding on curved roads, as displays of courage or strength. Some individuals merely use excessive speed to impress their friends”* (YL/31 years old)

#### 3.2.2 Theme 2: community perceptions of RTIs

Some people in the community view RTIs as a matter of fate. People who die or get injured on the roads are seen as suffering punishment for reasons known only to the victims. It is also perceived that the primary cause of accidents is young drivers who lack experience. Others regard RTIs as indicators of inadequate law enforcement, poor road conditions, and insufficient road signs and pavement markings. Some argue that the current methods for preventing road accidents are ineffective because they are based on Western ideas.

*“Many people drive unsafely; they speed or drive aggressively but do not get involved in accidents. I think those who are injured or die have their time. God planned for them to be injured or die that way.”* (PV / 41 years old)

*“Injuries or fatalities from road accidents serve as a form of punishment, and only the victims understand why.”* (CH/45 years old)

*“The lack of law enforcement, the poor state of road conditions, and the lack of signage or pavement markings on most of our roads often result in these RTIs.”* (PR / 58 years old)

#### 3.2.3 Theme 3: cultural and social norms

The participants highlighted cultural and social norms as factors that encourage unsafe driving behaviors. They perceived that cultural norms linking dangerous driving behaviors to gender, viewing driving as a male-only role, particularly among commercial drivers, make it challenging to address. Conversely, social norms that promote unsafe driving include the community’s acceptance of drinking and driving.

*“Because driving is seen as a male job, it would be challenging to get them to change, as acts of dangerous driving like speeding or drinking and driving serve as a way to prove their masculinity. Encouraging women to drive may make our roads safer”.* (YL 31 years old)

“R*oad safety programs must include cultural methods, such as teaching children at home who aspire to be drivers to respect all road users, especially women and children. Our current road safety activities will not be practical, as they are seen as Western ideas”.* (CH 47 years old)

*“Drivers can operate their vehicles safely if both passengers and the wider community take responsibility to call them out or report to the police when they witness unsafe driving behavior. The community perceives drinking and driving during celebrations as acceptable”.* (PO 36 years old)

## 4 Discussion

The study examined how gender roles and cultural beliefs influence risky driving habits in Vanuatu, providing local insights for road safety efforts and behavioral change programs. Three main themes emerged from the findings: Masculinity Norms, Community Perceptions of RTIs, and Cultural Norms as Barriers to Safe Driving. These themes demonstrate how traditional gender roles, fatalistic attitudes, and social permission to take risks create barriers to both safe driving and effective behavior change initiatives.

### 4.1 Masculinity norms surrounding dangerous driving behavior

The findings of this study suggest that cultural norms significantly influence not only how drivers behave but also who is deemed fit to drive. Driving is often viewed as a male-dominated profession in Vanuatu. When examining the impact of cultural values and traditions on driving in Vanuatu, the findings indicate that cultural beliefs restrict women from operating public transport and commercial vehicles. At the same time, participants consistently described risky driving, such as speeding, drink driving, or overtaking in hazardous conditions, as a way for men to demonstrate strength, courage, and social status, reinforcing the cultural view of driving as a masculine pursuit. Participants’ perceptions that women are weak, unable to protect themselves, and vulnerable to accidents exclude them from opportunities to drive commercial vehicles and public transport. This notion also suggests that men are dangerous drivers, and the roads would be safer if women were in charge of public and commercial driving. International evidence supports these insights. In a study conducted in Spain by Castro et al. to explore the truth behind these myths, the results show that Spanish women pay closer attention to traffic regulations and behave more cautiously, which should be considered when designing targeted preventive policies and road safety awareness strategies (39). Other studies indicate that men are more likely to take risks and, statistically, they are involved in more traffic accidents (39–41). Community-led road safety initiatives in Kenya and Pakistan have demonstrated that engaging local leaders and youth groups can effectively shift risk-related norms (13, 42). Integrating similar community-driven peer education and gender-inclusive programs could help challenge masculine risk-taking norms in Vanuatu.

### 4.2 Community perceptions of RTIs

Community perceptions play a critical role in shaping responses to RTIs. Many participants viewed crashes as acts of fate or divine will, while others viewed formal road-safety initiatives as “Western ideas” disconnected from local realities. They believed that injuries and deaths from road accidents occur because “it is their time” or as punishment for unknown transgressions. Such fatalistic beliefs reduce the perceived need for preventive measures and weaken support for enforcement efforts. This insight is consistent with studies in other parts of the world, including those in Latin America, which examine the impact of fatalistic beliefs and risk perceptions on road safety attitudes. The findings showed that those that reported more fatalistic beliefs also reported more dangerous attitudes to road safety and a lower perception of on-road risk (43). Another study conducted in Pakistan found that fatalistic beliefs are widespread, closely linked to religion, likely hinder the effectiveness of road safety messages, and contribute to risky road use (42).

Participants also pointed to inconsistent law enforcement, poor road conditions, and inadequate signage as contributing factors; however, the dominant narrative of inevitability undermines the urgency for change. These findings align with the broader literature on the influence of fatalism on road safety and underscore the challenge of encouraging preventive behavior when accidents are perceived as unavoidable (3, 6). Addressing these attitudes is essential to foster a sense of collective responsibility for safer driving.

### 4.3 Cultural norms as barriers to safe driving

The study also found that the community’s acceptance of drinking and driving encourages unsafe driving behavior. Participant narratives reveal that drivers can operate their vehicles safely if passengers and the wider community take responsibility by calling them out or reporting unsafe driving behavior to the police when they witness it. It is believed that normalizing drinking and driving during celebrations contributes to accidents and RTIs. This is consistent with the WHO Global Status Report on Road Safety, which indicates that traffic crashes rise during festive periods due to impaired judgment and risk-taking behavior. When communities accept and normalize actions such as speeding, drink-driving, and reckless driving during celebrations, road accidents and fatalities significantly increase (6). In their World Report on RTI Prevention, Peden et al. similarly emphasized that community tolerance for unsafe driving during celebrations diminishes the perceived importance of traffic laws (3). Drivers may feel emboldened to disregard safety measures, as they are aware that such violations are socially accepted during these events. This undermines long-term efforts in law enforcement (6, 7). Traffic psychologists are increasingly recognizing cultural beliefs and traditions as factors that influence driving behaviors and attitudes toward road safety (44). Vanuatu is a male-dominated society (45), where commercial driving and public transport roles are mainly seen as men’s work. Risky driving behaviors, such as speeding and overtaking, are often seen as expressions of strength and masculinity. The findings of this study indicated a cultural acceptance of male drivers who drink and drive during celebrations such as weddings and other social events. Because they are men, this behavior is generally tolerated and often normalized, making unsafe driving quite common. It is perceived that if women received the same respect as men to drive commercial and public transport vehicles, the roads would be safer. Oscar et al., in their study on the sex disparity in risky driving for Colombian drivers, found that Males consistently reported more dangerous driving behaviors, with about one-quarter of all participants indicating exposure to such behavior. Their findings showed that males experienced greater crash involvement, with violations such as speeding linked to crashes for both males and females (46). Other studies reaffirm gender as one of the most crucial explanatory factors for road crashes, which are the leading cause of death worldwide (40). The lingering question is whether these differences between males and females stem from biological factors or social constructs. In their study on the effect of culture on gender differences in risky driving behavior, Marie et al. demonstrate that gender differences vary based on the cultural context (40). This highlights the need to incorporate cultural considerations when developing road safety interventions.

## 5 Recommendations

The results of this study demonstrate that Vanuatu must develop road safety programs that challenge cultural norms contributing to dangerous driving. The programs should:

- Engage community and traditional leaders: Traditional leaders and community members collaborate to challenge fatalistic beliefs while ensuring everyone shares responsibility for road safety.
- Design gender-sensitive campaigns: Operationalizing these campaigns may include partnerships with transport associations, churches, and chief councils to co-design messages that emphasize responsibility, family safety, and gender inclusion.
- Integrate culturally resonant strategies: Combine culturally relevant methods such as visual prompts, faith-based outreach, and discussions to create enduring behavior change in the community.

These approaches will create behavior-change interventions that are both culturally appropriate and effective in achieving a successful reduction of dangerous driving and road traffic injuries in Vanuatu.

## 6 Conclusion

The study reveals that dangerous driving in Vanuatu is rooted in cultural factors that emphasize masculine norms, linking risky driving to notions of male strength and social status. Young men frequently speed and drink while driving to showcase their bravery, while their community perceives road accidents as fate and considers Western-style road safety measures ineffective. The absence of female commercial and public transport drivers limits opportunities for women to participate and become role models due to cultural and structural barriers. These findings indicate that reducing accidents and RTIs requires more than just physical infrastructure and law enforcement; it demands driver behavior change programs that align with local cultural values. To achieve sustainable road safety improvements in Vanuatu, strategies must challenge harmful gender stereotypes, involve community leaders and traditional figures, and encourage universal driving practices.

### 6.1 Study limitation

This study was conducted solely in Port Vila, which may limit how well the findings apply to rural Vanuatu. Additionally, all interviews were conducted in Bislama and later translated into English; although care was taken to preserve meaning, some linguistic nuances may have been lost during translation. The relatively small sample size, although enough for thematic saturation, might not reflect the full range of experiences across all regions of Vanuatu.

## Data Availability

The raw data supporting the conclusions of this article will be made available by the authors, without undue reservation.

## Appendix A: IDI & FGD guiding questions

I. Check relevant category

**Table:**

Traffic law enforcement officer	Police
	Land transport authority
	Municipal warden
Vehicle driver	Public transport drivers
	Commercial vehicle drivers
	Private vehicle drivers
Community leader	Chiefs
	Youth leaders
	Church leaders

II. Demographic questionnaire

1. How old are you?
2. Gender – M/F
3. What’s your highest level of education?
4. Do you drive? If yes, how long have you been driving?

III. Open-ended questionnaire

1. What do you think are the main causes of road accidents in your community?
2. How do men and women differ in their driving habits?
3. How do cultural beliefs or community expectations influence driving behavior?
4. In your opinion, what role does the community play in encouraging or preventing unsafe driving practices?
5. How do people in your community respond when someone drives dangerously or causes an accident?
6. What kinds of changes do you think are needed to make driving safer in your area?
